# Lessons learned from crises in blood donation services: A systematic review of operational practices

**DOI:** 10.1111/vox.70337

**Published:** 2026-08-20

**Authors:** Michael Garkisch, Stefanie Popp

**Affiliations:** ^1^ Faculty of Social and Health Sciences Technical University of Applied Sciences Regensburg (OTH Regensburg) Regensburg Germany; ^2^ Faculty of Economics and Social Sciences Helmut‐Schmidt University Hamburg Hamburg Germany; ^3^ Institute for Rescue Services, Emergency and Disaster Management (IREM) Technical University of Applied Sciences Würzburg‐Schweinfurt (THWS) Würzburg Germany

**Keywords:** blood donation services, blood supply, crisis management, disaster preparedness, operational practices, systematic review

## Abstract

**Background and Objectives:**

Blood donation services are vital to healthcare delivery, ensuring the continuous availability of safe and adequate blood products, particularly during periods of disruption and crisis. Disruptive events such as pandemics, mass‐casualty incidents, (natural) disasters, pandemics, armed conflicts and blood shortages can affect donor availability, logistics, workforce capacity, communication and operational continuity. This study systematically identifies and synthesizes lessons learned from crises affecting blood donation and translates them into a structured framework of operational practices.

**Materials and Methods:**

A systematic literature review between 2010 and 2025 was conducted using four databases. Study selection followed the Preferred Reporting Items for Systematic Reviews and Meta‐Analyses (PRISMA) guidelines. A total of 142 included publications were analysed using qualitative content analysis with MAXQDA and organized using an analytical framework adapted from the International Society of Blood Transfusion (ISBT) COVID‐19 Operational Recommendations Framework.

**Results:**

The analysis identified six overarching dimensions of operational practice in blood donation services: collaboration, coordination and standards; blood supply chain design and management; resource management; disaster preparedness and risk management; communication; and operations. Three overarching operational practice themes emerged: coordinated governance and communication, operational continuity and organizational resilience.

**Conclusion:**

Crises affecting blood donation services generate transferable lessons for maintaining safe and reliable blood supply operations under disruptive conditions. This review provides a structured synthesis of cross‐crisis operational practices and offers an evidence‐based foundation for blood services.

**Highlights**

- Here, we report the first systematic cross‐crisis review of lessons learned in blood donation services.
- The lessons learned are organized into six operational practice dimensions.
- Preparedness, operational continuity, coordinated response capabilities and organizational resilience are key for blood donation services facing disruptions.

## INTRODUCTION

Despite intensive research and advances, blood and blood products remain irreplaceable in modern healthcare due to their complexity and functional specificity [1, 2, 3]. Consequently, securing a continuous and safe blood supply represents a core responsibility of healthcare systems worldwide [4, 5, 6]. As also emphasized by the World Health Organization [7], blood donation services constitute a vital component of health system infrastructure and are widely recognized as part of the critical infrastructure sector [8, 9]. Their system relevance becomes especially evident during emergency and crisis situations, when blood demand can fluctuate unpredictably and supply chains are disrupted [8, 10, 11, 12].

Blood donation services have long been confronted with recurring disruptive events, such as (natural) hazards [11, 13], health emergencies like pandemics [14, 15, 16], armed conflicts [17], mass‐casualty incidents [18, 19], and financial crises [20]. These events can lead to increasing difficulties in donor recruitment and retention [21] and can create operational and logistical constraints, including blood shortages [22, 23], supply chain disruptions [24] and workforce shortages [22].

At the same time, crises not only reveal vulnerabilities in blood donation services but also generate valuable opportunities for organizational learning and improvement [25, 26, 27]. Lessons learned from previous disruptions provide valuable insights into the operational and managerial practices required to maintain service continuity under challenging conditions [28, 29, 30]. Systematically identifying and synthesizing these experiences can help organizations strengthen preparedness, improve response capabilities and support continuity of service during future disruptions [31, 32, 33, 34].

While research on crises affecting blood donation services has expanded considerably, lessons learned remain fragmented across individual events and disciplines. As a result, transferable operational practices and organizational responses have not yet been systematically synthesized. Therefore, this study conducts a systematic literature review (SLR) to identify and organize cross‐crisis lessons learned into a structured framework of operational practices (adapted from [35]).

## MATERIALS AND METHODS

### Study design and review aim

Existing research on crises affecting blood donation services primarily focuses on individual events, leaving operational lessons learned fragmented across crisis contexts. Therefore, this study systematically explores and consolidates evidence from crises affecting blood donation services. SLRs are well suited to provide an evidence‐based overview of current knowledge, identify research gaps and support conceptual development [36, 37, 38].

### Research questions and analytical framework

The review is guided by two research questions (RQ1/RQ2). To facilitate systematic analysis and comparison across studies, an analytical framework adapted from the ISBT Operational Recommendations Framework [35] was adopted. The ISBT framework was selected because it provides an internationally recognized operational classification specifically developed for blood donation services.

*Which lessons learned from crises affecting blood donation services are reported in scientific literature?*

*Which overarching operational practice themes emerge from the synthesis of cross‐crisis lessons learned in blood donation services?*

### SLR methodology—Search strategy and data sources

A structured search strategy combining crisis‐related (topic) and blood donation‐related (sector) keywords (see Table S1) was applied across four databases, namely EBSCO Business Source Premier, ABI/INFORM, Scopus, and PubMed, and cross‐checked using Google Scholar, consistent with established methodological approaches [36, 37, 38] and previous reviews in the field [39, 40, 41].

### Eligibility criteria and study selection

Study selection followed predefined inclusion and exclusion criteria (see Table S2). Publications were required to address crises, disasters, resilience, robustness or disruptions affecting blood donation services. Only peer‐reviewed articles published in English between 2010 and 2025 were included. Two researchers independently assessed all publications during the title, abstract, and full‐text screening stages. Divergent assessments were addressed through discussion until agreement was reached.

### Data extraction and qualitative content analysis

An in‐depth qualitative content analysis was performed on the final sample [42, 43] of 142 publications. Using MAXQDA, lessons learned and operational practices were extracted and synthesized within the adapted analytical framework [35]. Across several iterative coding cycles, more than 700 coded text segments were compared, refined and consolidated into higher‐order attributes; ambiguous cases and coding disagreements were discussed between the researchers and resolved by consensus. The dimensions were largely aligned with the ISBT framework, while the corresponding attributes were inductively derived from the literature to structure the findings (see Table S3). Given the heterogeneous nature of the included literature and the study's focus on identifying cross‐crisis operational lessons learned, no formal methodological quality assessment was performed.

### Reporting standards

The review process was conducted and reported in accordance with the PRISMA guidelines for systematic reviews [44, 45]; see Figure S1.

## RESULTS: LESSONS LEARNED DIMENSIONS AND ATTRIBUTES

To address RQ1, the identified lessons learned were synthesized and organized into six dimensions and corresponding attributes derived through qualitative content analysis. Figure 1 provides an overview of the analytical framework used to structure the findings.

**FIGURE 1:**
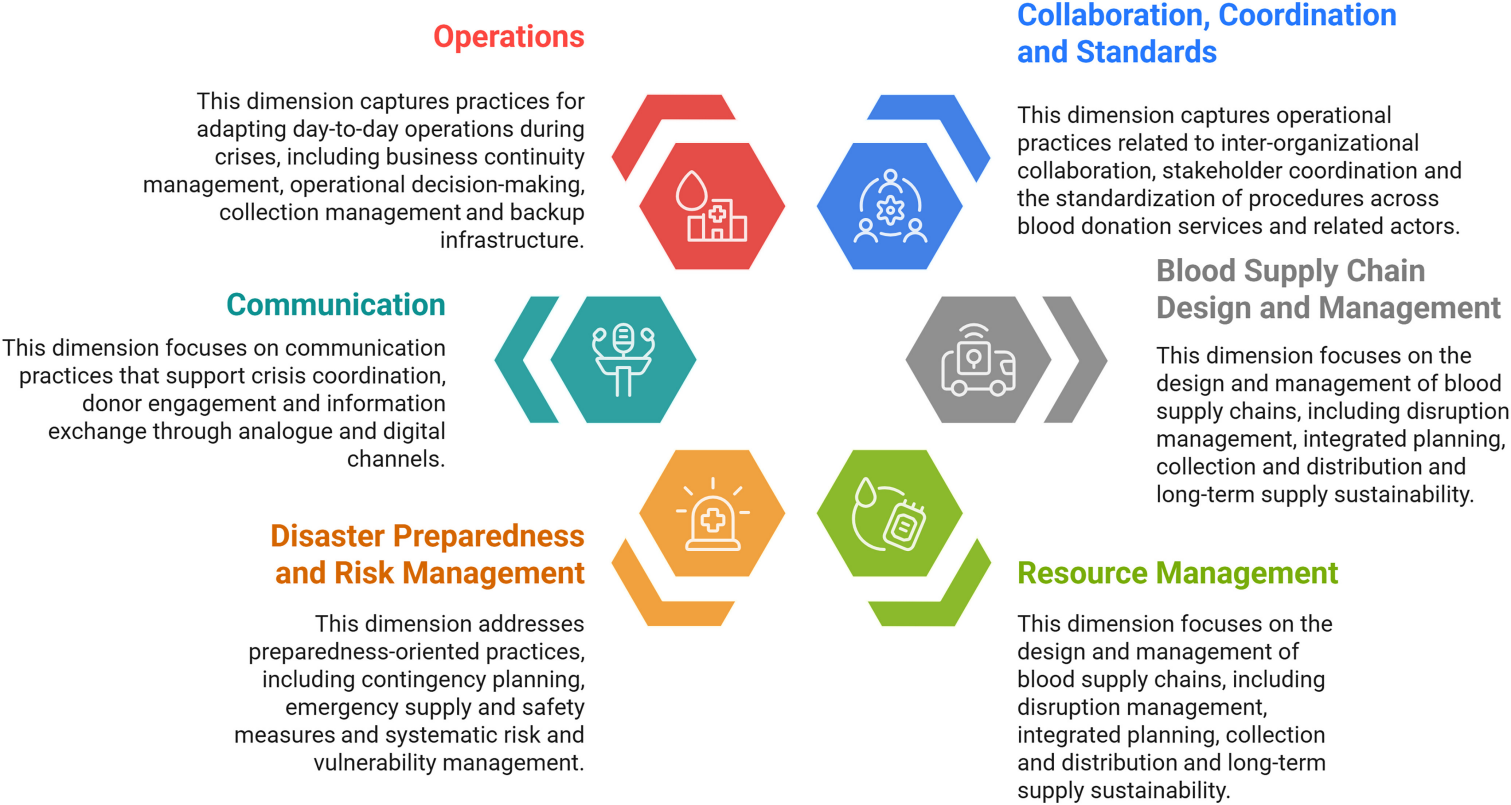
Overview of the six operational practice dimensions for blood donation services and their core characteristics.

For each of the six dimensions and their associated attributes, we developed practice‐oriented tables that translate lessons learned into actionable operational recommendations. These tables are provided as Tables [Link], [Link].

### Collaboration, coordination and standards

#### Collaboration and knowledge sharing

Across crisis contexts, collaboration and knowledge sharing consistently emerged as recurring lessons learned in blood donation services [46]. Evidence from previous crises suggests that successful collaboration was supported by structured communication and coordination mechanisms [35, 47], transparency and trust among participating organizations [48] and the ability to adapt interaction and information‐sharing practices as crisis situations evolved [49].

#### Multi‐stakeholder coordination

Coordinated action among blood donation services, public authorities, suppliers and clinical teams repeatedly emerged as an important operational practice during crises [35]. Several studies report that formalized coordination structures with clearly defined roles and responsibilities supported preparedness and response activities, particularly during infectious disease outbreaks [50]. Effective stakeholder coordination was also associated with improved resource allocation when capacities were constrained [51].

#### Integrated blood donation service systems

Experiences from disasters and other crises consistently highlight the importance of coordinated blood management across national, regional and local levels. National coordination mechanisms supported the planning of blood production, distribution and emergency response activities [50]. Similarly, nationally coordinated blood systems facilitated the efficient matching of supply and transfusion demand during crisis situations, thereby reducing supply gaps [52]. A recurring lesson across studies was the importance of integrating regional blood centres and local hospitals into blood management [53].

#### Standardized practices and procedures

Another recurring lesson learned concerned the importance of standardized practices and procedures during crises. Across multiple studies, harmonized guidelines were reported to improve consistency and operational efficiency under conditions of uncertainty and time pressure [35]. Standardized procedures provided a common basis for blood supply management and emergency response activities [50, 54], while also supporting more coordinated approaches to blood distribution and utilization [55]. In addition, several studies highlighted the value of standardized inventory management practices for maintaining oversight and supporting decision‐making during periods of disruption [56, 57].

### Blood supply chain design and management

#### Uncertainty and disruption management

Across different hazards and disaster contexts, blood supply chains were consistently characterized by high levels of uncertainty. Studies report considerable volatility in both supply and demand, driven by disrupted donation patterns [35, 48, 58], infrastructural damage [52, 59, 60] and rapidly changing clinical requirements [61, 62, 63]. Evidence across crises indicates that insufficient anticipation of such disruptions contributed to shortages [13, 35, 64], delivery challenges [59, 65] and inefficient allocation of blood products [66, 67]. A recurring operational practice therefore involved improving visibility, forecasting and disruption management across the supply chain [52, 68]. Several studies additionally describe the use of technology‐enabled solutions, including drones [59], artificial intelligence for forecasting [69] and blockchain applications [70].

#### Integrated and adaptive blood supply chains

A recurring finding across crisis situations is the importance of managing the blood supply chain as an integrated and adaptive system. Disruptions at any stage of the chain can have immediate consequences for patient care and healthcare operations [68, 71, 72]. The studies consistently identify the maintenance of a sufficient and reliable supply of blood products as the key operational objective [73]. Several publications highlight the strong interdependencies between donor populations [67, 74], permanent blood centres [67, 74, 75], mobile collection facilities [66, 76], processing facilities and distribution networks [64, 77]. To maintain operational continuity during disruptions, evidence consistently emphasizes the need for integrated planning [61].

#### Blood collection and distribution management

Blood collection and distribution repeatedly emerged as particularly vulnerable operational processes during crises. Studies report that centralized collection and distribution models were often strained when transportation routes, facilities or workforce capacities were disrupted [53]. Consequently, decentralized collection structures and reliable blood centre networks were frequently identified as effective operational practices [52, 66, 78]. Effective management of collection and distribution activities was consistently associated with the consideration of disruption risks [66, 79, 80] and the capabilities of available transportation options [81, 82]. Combining different transportation modes helped reduce delays when infrastructure was impaired [74]. Several studies also underline the importance of actively managing both demand [46, 52, 71, 83] and supply [50, 83]. During shortages, blood stewardship practices supported a more targeted use of scarce blood products [84]. Operational responses further included extended opening hours [85], additional donation sites [59], mobile collection units [66, 76] and digital appointment systems to improve donor flow management and planning reliability [86].

#### Long‐term planning and supply sustainability

Beyond immediate response measures, the literature highlights the importance of long‐term planning to ensure sustainable blood supply operations [87, 88]. Crisis situations frequently amplified existing challenges associated with the perishable nature of blood products, leading either to increased wastage [56, 89] or severe shortages [66, 89, 90, 91]. Across studies, improved inventory management, forecasting approaches and demand‐planning tools were identified as important practices for supporting supply sustainability [88, 91]. Emerging technologies, including artificial intelligence for demand forecasting, were also discussed as mechanisms for improving longer‐term planning capabilities [69].

### Resource management

#### Workforce protection, support and training

Across crisis contexts, workforce availability consistently emerged as a critical factor affecting the operational performance of blood donation services [35, 49]. Staff shortages frequently increased workloads for remaining personnel [35] and created bottlenecks in blood collection and processing activities [92]. Another study reports that workforce disruptions substantially reduced operational capacity and intensified pressure on healthcare systems [92]. A recurring lesson across crises was the need to strengthen workforce protection and support mechanisms before disruptions occur. This included integrating psychological support and crisis intervention measures into contingency planning [93], providing trauma support for affected personnel [94]. Training also emerged as an important operational practice. Studies highlight the need for rapidly deployable training programmes during crisis situations [95]. During the COVID‐19 pandemic, practical instruction on infection prevention measures, including social distancing, surface and hand hygiene, and respiratory protection, supported safe operational continuity [86]. In addition, workforce environments that encouraged flexibility, problem‐solving and adaptive working practices were better able to respond to changing operational demands [49, 94].

#### Inventory management and monitoring

Managing blood inventories under volatile conditions emerged as a recurring operational challenge across crisis contexts. Maintaining situational awareness of blood availability and usage was often complicated by rapidly changing demand patterns [85]. Consequently, real‐time monitoring systems were frequently described as an important practice for identifying shortages and imbalances at an early stage [91]. Several studies report the implementation of digital inventory systems and blood‐sharing mechanisms to support regional and local balancing of blood supplies [91]. At a broader level, nationally coordinated inventory management systems were associated with improved oversight and coordination of available resources [96]. During shortages, prioritization of blood products for critical patients and the use of alternative treatment options were frequently reported operational responses [93]. Beyond product inventories, several studies highlighted the value of monitoring donor behaviour and donation patterns [86, 97].

#### Decision support systems and scenario analysis

A recurring finding across crises was the need to support operational decision‐making under conditions of significant supply and demand uncertainty. Several studies approached blood supply management as a decision‐support challenge, enabling more structured responses to changing circumstances [98]. Scenario‐based planning, sensitivity analyses and optimization models were frequently applied to evaluate the effects of changing demand levels, inventory capacities, operating times and transportation constraints [90, 99]. The literature further underscores the need for continuous learning and adaptation in organizational decision processes. Iterative review, organizational learning and adjustment of operational strategies supported more effective responses during ongoing disruptions [54, 100]. Strategic planning was likewise identified as an essential component of effective blood supply management [61]. Studies additionally note that maintaining adequate blood availability often required combining operational planning measures with actions aimed at sustaining donor participation [91].

#### Donor recruitment strategies

Maintaining donor participation emerged as a recurring challenge across multiple crisis contexts, particularly during infectious disease outbreaks and periods of heightened public uncertainty [95]. Several studies indicate that disruptions were influenced not only by logistical constraints [52, 80], but also by donor concerns regarding safety [48], infection risks [35, 48] and confidence in donation processes [101, 102]. To address these challenges, blood donation services implemented a range of recruitment‐related practices, including strengthening donor programmes [15, 49, 103], actively managing public perceptions [48, 104] and expanding donor communication efforts. Proactive donor education was repeatedly associated with increased willingness to donate during crisis situations [48]. Recruitment approaches commonly targeted both altruistic and self‐interested motivations, emphasizing the lifesaving value of donation while also addressing personal concerns and incentives [103]. Several studies additionally highlight the importance of broadening the donor base beyond traditional donor groups to compensate for crisis‐related declines in participation [84], like healthcare employees [48].

#### Donor retention and engagement

While recruiting new donors was an important operational objective during crises, retaining existing and newly acquired donors emerged as an equally important challenge. Several studies indicate that maintaining a stable blood supply depended not only on donor recruitment but also on converting first‐time crisis donors into repeat donors [97, 105]. Evidence across studies suggests that positive donation experiences increased the likelihood of repeat donation and long‐term engagement [49, 58]. Beyond individual experiences, donor retention was closely linked to social relationships, collective norms and perceptions of social responsibility [103]. Experiences from the COVID‐19 pandemic further highlighted the dependence of blood donation services on the willingness of healthy donors to contribute to the public good [46]. Across crisis contexts, donor motivations proved to be multifaceted and dynamic [103].

### Disaster preparedness and risk management

#### Contingency and disaster planning

Sustaining a reliable and secure blood supply during emergencies depends heavily on effective contingency and disaster planning. Across different crisis types, including infectious disease outbreaks and natural disasters, studies repeatedly report that the unpredictable nature of crises exposed weaknesses in existing planning arrangements [49, 95, 106]. Evidence across crises indicates that planning activities were most effective when risk assessments, vulnerability analyses, mitigation measures and contingency plans were embedded in routine blood donation service management rather than developed reactively during emergencies [46, 106]. Several studies further highlight that unclear roles [54], responsibilities [54] and escalation pathways [46] complicated coordination and delayed operational responses during the early stages of crises. Effective emergency preparedness also requires integrated logistics planning and clearly defined response structures [107, 108]. A recurring operational practice was the integration of blood centres into broader local and national emergency response systems. Studies show that blood donation services were better able to assess emerging needs and coordinate response activities when they were explicitly incorporated into local and national emergency planning structures [50, 95]. Several publications additionally emphasize the importance of regularly reviewing and updating contingency plans. Plans that were outdated or insufficiently operationalized often provided limited guidance under rapidly changing conditions [35, 92]. Effective plans commonly included predefined transfusion prioritization criteria, guidance for blood rationing during shortages [54] and alignment with hospital‐level emergency planning arrangements [18]. Furthermore, proactive leadership and crisis management structures across the blood supply system were frequently identified as important enablers of effective emergency response [96].

#### Maintaining blood supply and safety during emergencies

A consistent finding across crises was the need to implement operational measures that maintain blood availability while ensuring product safety. One frequently reported practice was the establishment of buffer stocks to compensate for anticipated or ongoing disruptions [56, 109]. Studies also describe the use of alternative supply arrangements, including inter‐centre cooperation, resource‐sharing mechanisms and mutual support agreements to address localized shortages [52, 61, 71, 110]. Flexible sourcing approaches, such as mobile collection facilities, were similarly used to maintain blood availability when conventional collection structures were constrained [59, 64, 111]. Blood safety emerged as an equally important operational priority. Several studies report the introduction of additional safety procedures to manage emerging biological risks and maintain confidence in the safety of blood products during crises [40, 49]. To counteract crisis‐related declines in donation activity, blood donation services frequently implemented complementary measures such as expanded mobile blood drives, targeted communication campaigns and coordination mechanisms for sharing available blood resources across organizations [91].

#### Risk and vulnerability management

Across crisis contexts, effective blood supply management was closely linked to the systematic identification and assessment of operational risks and vulnerabilities. Frequently reported risk factors included uncertainty in supply and demand [57, 88, 112], the perishable nature of blood products [56, 99] and vulnerabilities related to infrastructure and facility design [71, 113]. Several studies describe the application of analytical models, optimization approaches and risk‐based planning tools to support decision‐making under uncertain conditions [60, 114]. Beyond analytical methods, the literature highlights a range of proactive mitigation practices aimed at reducing identified risks. Examples include revised donor deferral criteria, enhanced surveillance procedures and notification systems designed to limit transfusion‐related infectious risks posed by emerging pathogens [49]. Overall, evidence across studies suggests that systematic risk assessment, vulnerability analysis and targeted mitigation measures form essential components of effective blood supply management during crises and large‐scale emergencies [13, 51, 106].

### Communication

#### Crisis communication

Studies consistently highlight the importance of structured crisis communication for managing information flows and stakeholder coordination during emergencies affecting blood donation services. Several publications report that the establishment of crisis committees provided a focal point for coordinating communication activities and disseminating information during crises [46]. Evidence across crises further indicates that effective communication extended beyond organizational boundaries and involved emergency response agencies and other stakeholders to support coordinated action and maintain blood supply operations [95]. A recurring finding was the importance of distinguishing between internal and external communication processes. Internally, communication among blood donation services [115] and between leadership and staff [58], supported operational alignment, role clarity and workforce engagement during rapidly changing situations [58]. Externally, communication with governments, regulatory authorities and other key stakeholders facilitated information exchange, decision‐making and resource mobilization [47]. Several studies additionally emphasize that communication approaches needed to be adapted to the specific characteristics of different crisis situations [20, 61].

#### Donor communication

Donor communication emerged as a critical operational practice across crisis contexts. Maintaining public awareness of the ongoing need for blood donations was often more challenging during emergencies, particularly when public attention shifted toward competing health risks or safety concerns [97]. During pandemics, concerns regarding personal health risks associated with donation were repeatedly identified as barriers to donor participation [15, 97]. Several studies report that targeted and coordinated communication measures helped address these concerns. Tailored media campaigns emphasizing altruistic motivations were frequently associated with successful donor mobilization during crisis periods [85]. In addition, clear and consistent communication regarding blood demand reduced uncertainty and supported continued donor participation [48, 85, 116]. A further recurring practice involved proactively communicating infection prevention and safety measures. Blood donation services that clearly communicated their adherence to recommended safety protocols were better able to reassure donors and sustain donation activity during crises [48]. Several studies further emphasize the need to clearly communicate the purpose of donation appeals and communicating the societal value and lifesaving impact of blood donation to support donor understanding and engagement during and beyond crisis periods [103, 104].

#### Digital communication strategies

Across crisis contexts, digital communication tools increasingly emerged as practical mechanisms for maintaining information exchange, donor outreach and organizational coordination under disrupted conditions. Several studies describe the use of targeted digital campaigns [85], social media communication [84, 97] and online donor communities [58] to reach potential donors when traditional communication channels were constrained. Internally, digital platforms supported information sharing and coordination among blood donation services. Centralized communication and collaboration systems were used to distribute updates, coordinate activities and support staff operating in rapidly changing environments [13, 117]. In addition, digital technologies facilitated training and competency development when face‐to‐face instruction was not feasible, thereby supporting the continuity of operational processes during crises [35, 82].

### Operations

#### Business continuity management

A recurring finding across crisis contexts was that routine management structures were often insufficient to cope with the operational demands of emergencies affecting blood donation services. Several studies report that the establishment of crisis committees with direct involvement of senior management became a central operational practice during crises [46]. In many cases, decision‐making shifted from routine administrative procedures to structured response processes specifically designed to support rapid operational adaptation under changing conditions [18]. Studies further highlight that the role of blood centres frequently evolved during crises. Local blood donation services often assumed responsibility as operational coordination hubs, facilitating information exchange, situational awareness and response coordination, particularly in disaster‐affected areas [118]. Overall, the literature indicates that maintaining continuity of operations often requires temporary adjustments to governance structures, decision‐making responsibilities and operational roles.

#### Blood collection and operational management

Several studies describe blood collection and operational management as key areas requiring adaptation during and after crises. One frequently reported challenge concerned sudden surges of donors following high‐impact events. While increased donor willingness was generally beneficial, uncoordinated donation peaks often created operational bottlenecks and capacity constraints at collection sites [95, 119]. Managing donor surges and converting first‐time donors into repeat donors therefore emerged as a recurring operational challenge [102, 105]. Another operational concern involved managing the perishability of blood products while maintaining adequate availability [112, 120]. Crisis analyses highlight effective management of blood perishability and minimizing wastage and ensuring timely delivery of blood products [120]. Several studies report the application of integrated operational approaches spanning multiple stages of the blood supply process during disruptions [66, 77, 108]. Examples included centralized hospital inventory management to reduce wastage and alleviate bottlenecks [57], maintaining safety stocks to buffer acute disruptions [109] and considering alternative treatment options [93].

#### Operational decision‐making under uncertainty

Operational decision‐making during crises was repeatedly characterized by high levels of uncertainty regarding demand, capacity, facility availability and resource requirements [87]. Several studies report that conventional planning assumptions were often insufficient to capture the volatility observed during emergencies [87]. Consequently, robust and flexible planning approaches were used to support operational decision‐making under uncertain conditions [53]. A recurring operational practice involved the use of real‐time information to support rapid adjustment of activities. Monitoring systems that provided timely information on supply, demand and logistics enabled more responsive operational management and facilitated adjustments as conditions evolved [91].

#### Facility accessibility and backup infrastructure

Facility accessibility and infrastructure planning emerged as important operational factors during crises. Several studies report that the strategic location of blood collection and processing facilities supported service continuity and emergency response activities [73]. Additional or geographically closer donation sites were particularly valuable during pandemics, when travel restrictions or safety concerns affected donor mobility [120]. The literature further highlights the importance of backup facilities and contingency infrastructure. Alternative collection sites and redundant facility networks helped compensate for outages, access restrictions or disruptions affecting primary locations [120]. Several studies also describe the adaptation of traditional collection approaches through measures [46, 96] such as appointment systems, telephone‐based health screening and coordinated contingency arrangements with partner organizations [46].

## DISCUSSION: FROM LESSONS LEARNED TO OPERATIONAL PRACTICE THEMES

### Cross‐crisis synthesis—Overarching operational practice themes

To address RQ2, the six dimensions and their associated attributes were analysed at a higher level of abstraction to identify recurring cross‐crisis patterns of operational practice. While the six dimensions and associated attributes capture specific operational practices reported across crisis contexts, a cross‐dimensional synthesis reveals three overarching operational practice themes that consistently shaped the ability of blood donation services to respond to and operate during crises:

1. *Coordinated governance and communication* emerged as a central operational practice, as effective crisis response depended on structured collaboration, clearly defined roles, harmonized standards and reliable information flows between blood donation services, authorities, hospitals, suppliers, staff and donors [35, 46, 48, 50, 95].
2. *Operational continuity* was primarily supported by adaptive supply chain, resource and decision‐making practices, including integrated planning, flexible collection and distribution structures, inventory monitoring, forecasting, scenario analysis and real‐time adjustment of operational processes under uncertainty [52, 66, 68, 90, 91, 98].
3. *Organizational resilience* was strengthened when preparedness, risk management, workforce protection, donor recruitment and retention, and systematic learning from previous crises were institutionalized before disruptions occurred rather than improvised during acute events [13, 49, 54, 94, 97, 106].

### Limitations

As with any SLR synthesising existing knowledge, this study is subject to limitations arising from the search strategy, the restriction to peer‐reviewed English‐language publications, and the exclusive reliance on secondary data. Therefore, despite iterative search refinement and citation tracking, some relevant studies may not have been identified. Furthermore, the review adopts a broad cross‐crisis perspective that supports the identification of recurring operational practices but limits the attribution of findings to specific crisis types, healthcare systems or governance contexts. In addition, the heterogeneous nature of the literature included precluded a formal methodological quality assessment; therefore, the findings should be interpreted as a synthesis of recurring operational practices rather than an appraisal of the strength of evidence underlying individual recommendations.

### Conclusion

Crises affecting blood donation services generate transferable lessons that extend beyond individual events. Through a cross‐crisis synthesis of the literature, six operational practice dimensions and three overarching operational practice themes were identified. Together, they provide a structured framework for strengthening preparedness, operational continuity, coordinated response capabilities and organizational resilience in blood donation services facing future disruptions.

## CONFLICT OF INTEREST STATEMENT

The authors declare no conflicts of interest.

## Supporting information


**Figure S1.** PRISMA flow diagram illustrating the identification, screening, eligibility assessment and inclusion of studies in the systematic literature review.

**Table S1.** Crisis‐related and blood service‐related keywords used for systematic literature search.

**Table S2.** Overview of the inclusion and exclusion criteria.

**Table S3.** Adaptation and expansion of the ISBT Operational Recommendations Framework [35] into six dimensions of operational practice: Analytical scope, delineation and interdependencies.

**Table S4.** Dimension 1—Collaboration, coordination and standards: Attributes and practical recommendations.

**Table S5.** Dimension 2—Blood supply chain design and management: Attributes and practical recommendations.

**Table S6.** Dimension 3—Resource management: Attributes and practical recommendations.

**Table S7.** Dimension 4—Disaster preparedness and risk management: Attributes and practical recommendations.

**Table S8.** Dimension 5—Communication: Attributes and practical recommendations.

**Table S9.** Dimension 6—Operations: Attributes and practical recommendations.

## Data Availability

The data underlying this study comprise: (i) a comprehensive Excel file documenting the complete database search strategy and search process, (ii) a bibliography of all publications included in the qualitative content analysis and (iii) the corresponding coding framework and coded dataset generated using MAXQDA. The Excel overview, literature list and MAXQDA coding files are not publicly available due to their nature and content but are available from the corresponding author upon reasonable request.
