# Supplementary material for: Lessons learned from crises in blood donation services: A systematic review of operational practices
Source: Vox Sang. 2026 Aug 20;121(10):1553–64. doi: 10.1111/vox.70337 (PMC13649367; doi:10.1111/vox.70337)
Supplement: Supplementary file 1 [file VOX-121-1553-s001.pdf]

Supplementary Table 6: Dimension 3 – Resource Management: Attributes and Practical Recommendations

Dimension	Attribute	Practical Recommendation	Attribute	Practical Recommendation	Attribute	Practical Recommendation
**Dimension 3: RESOURCE MANAGEMENT**	**Workforce Protection, Support and Training**	1. Implement comprehensive workforce protection and support programs	**Decision Support Systems and Scenario Analysis**	1. Integrate decision-support systems into blood supply management	**Donor Retention and Engagement**	
		2. Establish rapidly deployable crisis training programs		2. Institutionalize scenario planning, sensitivity analyses, and optimization models		
		3. Foster flexible and adaptive work environments		3. Embed continuous learning and adaptive decision-making processes into strategic planning		1. Develop targeted retention strategies for first-time crisis donors
	**Inventory Management and Monitoring**	1. Implement real-time inventory monitoring systems	**Donor Recruitment Strategies**	1. Implement proactive donor education and communication programs		2. Enhance the donor experience throughout the donation process
		2. Establish digitally supported inventory-sharing and coordination mechanisms		2. Develop recruitment campaigns that combine altruistic and personal motivations		3. Foster long-term donor relationships by addressing diverse donor motivations and reinforcing social responsibility
		3. Integrate inventory monitoring with demand prioritization and donor monitoring activities		3. Broaden and diversify the donor base beyond traditional donor groups		
