# Supplementary material for: Lessons learned from crises in blood donation services: A systematic review of operational practices
Source: Vox Sang. 2026 Aug 20;121(10):1553–64. doi: 10.1111/vox.70337 (PMC13649367; doi:10.1111/vox.70337)
Supplement: Supplementary file 2 [file VOX-121-1553-s002.pdf]

Supplementary Table 7: Dimension 4 – Disaster Preparedness and Risk Management: Attributes and Practical Recommendations

	**Attribute**	Practical Recommendation	**Attribute**	Practical Recommendation
**Dimension 4: DISASTER PREPAREDNESS AND RISK MANAGEMENT**	**Contingency and Disaster Planning**	1. Integrate contingency and disaster planning into routine operations	**Risk and Vulnerability Management**	
		2. Establish and regularly update comprehensive emergency response plans		
		3. Strengthen integration with local and national emergency response systems		1. Implement systematic risk and vulnerability assessment frameworks across the blood system
	**Maintaining Blood Supply and Safety During Emergencies**	1. Establish buffer stocks and alternative supply arrangements		2. Integrate risk-based planning tools, analytical models, and optimization approaches into decision-making processes
		2. Develop flexible collection and sourcing capabilities		3. Establish proactive mitigation measures and surveillance mechanisms
		3. Implement enhanced safety and continuity measures during crises		
