# Supplementary material for: Lessons learned from crises in blood donation services: A systematic review of operational practices
Source: Vox Sang. 2026 Aug 20;121(10):1553–64. doi: 10.1111/vox.70337 (PMC13649367; doi:10.1111/vox.70337)
Supplement: Supplementary file 3 [file VOX-121-1553-s003.pdf]

Supplementary Table 4: Dimension 1 – Collaboration, Coordination and Standards: Attributes and Practical Recommendations

	Attribute	Practical Recommendation	Attribute	Practical Recommendation
**Dimension 1: COLLABORATION, COORDINATION & STANDARDS**	**Collaboration and Knowledge Sharing**	1. Establish formal collaboration and knowledge-sharing networks	**Integrated blood donation service systems**	1. Establish nationally coordinated blood management structures
		2. Implement structured communication and coordination mechanisms		2. Implement coordinated systems for balancing blood supply and transfusion demand
		3. Promote transparency, trust, and adaptive information-sharing practices		3. Integrate regional blood centres and local hospitals into blood management and planning processes
	**Multi-Stakeholder Coordination**	1. Establish formal multi-stakeholder coordination structures	**Standardized Practices and Procedures**	1. Develop and implement harmonized guidelines and standard operating procedures
		2. Define clear roles, responsibilities, and escalation pathways		2. Standardize blood supply management, distribution, and utilization processes
		3. Use stakeholder coordination to support resource allocation under constrained conditions		3. Establish standardized inventory management practices
