# Supplementary material for: Lessons learned from crises in blood donation services: A systematic review of operational practices
Source: Vox Sang. 2026 Aug 20;121(10):1553–64. doi: 10.1111/vox.70337 (PMC13649367; doi:10.1111/vox.70337)
Supplement: Supplementary file 4 [file VOX-121-1553-s004.pdf]

Supplementary Table 9: Dimension 6 – Operations: Attributes and Practical Recommendations

Dimension	Attribute	Practical Recommendations	Attribute	Practical Recommendations
**Dimension 6: OPERATIONS**	**Business Continuity Management**	1. Establish crisis management structures with direct senior leadership involvement	**Operational Decision-Making Under Uncertainty**	1. Adopt flexible and robust planning approaches
		2. Implement structured crisis response processes that complement routine management systems		2. Integrate real-time monitoring systems into operational decision-making
		3. Designate blood centres as operational coordination hubs during crises		3. Embed adaptive decision-making processes within crisis operations
	**Blood Collection and Operational Management**	1. Establish coordinated donor surge management procedures	**Facility Accessibility and Backup Infrastructure**	1. Strategically locate and expand blood collection and processing facilities
		2. Implement operational measures that address blood product perishability		2. Establish backup facilities and contingency infrastructure
		3. Apply integrated operational management approaches across the blood supply process		3. Implement adaptable collection and access procedures
