# Supplementary material for: Lessons learned from crises in blood donation services: A systematic review of operational practices
Source: Vox Sang. 2026 Aug 20;121(10):1553–64. doi: 10.1111/vox.70337 (PMC13649367; doi:10.1111/vox.70337)
Supplement: Supplementary file 5 [file VOX-121-1553-s005.pdf]

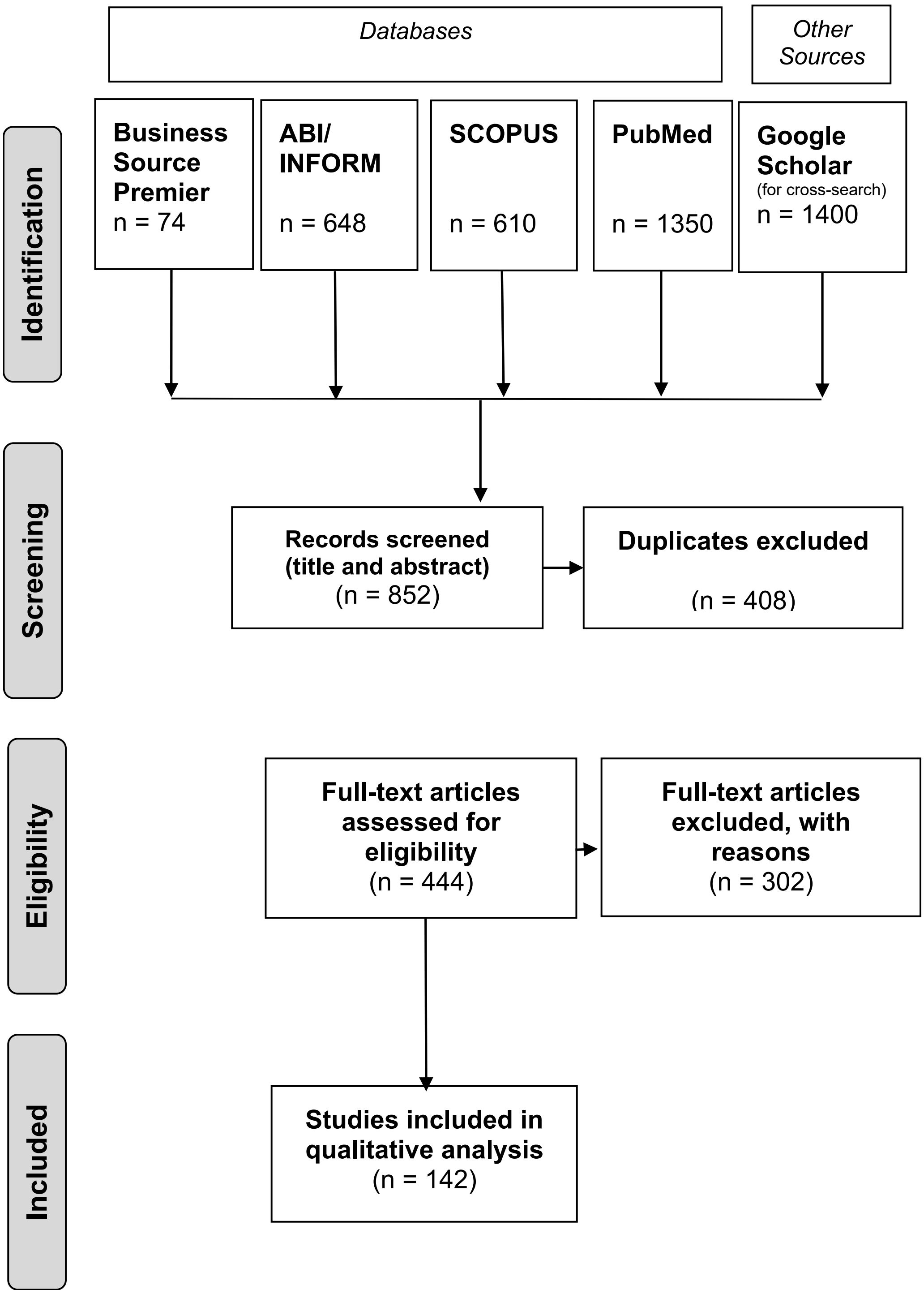

Supplementary Figure 1: PRISMA flow diagram illustrating the identification, screening, eligibility assessment, and inclusion of studies in the systematic literature review.
