# Supplementary material for: Lessons learned from crises in blood donation services: A systematic review of operational practices
Source: Vox Sang. 2026 Aug 20;121(10):1553–64. doi: 10.1111/vox.70337 (PMC13649367; doi:10.1111/vox.70337)
Supplement: Supplementary file 6 [file VOX-121-1553-s006.pdf]

Supplementary Table 3: Adaptation and Expansion of the ISBT Operational Recommendations Framework [35] into six Dimensions of Operational Practice: Analytical Scope, Delineation, and Interdependencies

**"Recommendations of International Society of Blood Transfusion survey of experiences of blood banks and transfusion services during the COVID-19 pandemic"** [35]	**Adapted and expanded: 6 Dimensions of Operational Practice**	**Primary Analytical Focus**	**Delineation and Interdependencies**
Operations	Dimension 6: Operations	Day-to-day and crisis-time operational execution	Distinct from Disaster Preparedness and Risk Management, which focuses on anticipatory planning and risk reduction. Operations focuses on the execution, adaptation, and management of day-to-day and crisis-related activities. Operational performance depends on capabilities developed within all other dimensions, including governance structures, supply chain design, resource availability, preparedness arrangements, and communication processes.
Protocols	Dimension 4: Disaster Preparedness and Risk Management	Preparedness, risk reduction, and anticipation	Distinct from Operations, which focuses on crisis execution and response. Closely interrelated with all other dimensions, as preparedness, contingency planning, and risk assessment provide the foundation for coordinated governance, communication, resource allocation, and operational continuity.

Resources →	Dimension 3: Resource Management	Management of critical organizational resources	Distinct from Supply Chain Management, which focuses on system structures and flows. Closely linked to Operations, Communication, and Disaster Preparedness, as workforce, inventories, donors, and decision-support capabilities enable operational continuity and organizational resilience.
Communications →	Dimension 5: Communication	Information exchange and stakeholder engagement	Distinct from Collaboration, which focuses on governance arrangements and stakeholder structures. Strongly interdependent with Collaboration, Resource Management, and Operations, as effective communication supports coordination, donor engagement, workforce alignment, and crisis response activities.
Collaboration →	Dimension 1: Collaboration, Coordination & Standards	Governance, coordination, and standardization	Distinct from Communication, which focuses on information exchange processes. Strongly interdependent with Communication and Disaster Preparedness, as effective coordination and standardized governance structures enable aligned crisis communication, preparedness planning, and system-wide response activities.
	Dimension 2: Supply Chain (New)	Supply chain resilience, design, and continuity	Distinct from Resource Management, which focuses on managing individual resources. Strongly interdependent with Operations and Resource Management, as supply chain performance depends on resource availability, operational execution, and coordinated decision-making across the network.

The analytical framework was adapted and extended from the ISBT Operational Recommendations Framework [35], which was derived primarily from experiences during the COVID-19 pandemic. While the original framework provided an important operational classification for blood donation services, the cross-crisis synthesis conducted in this review revealed several recurring operational challenges that extended beyond the scope of pandemic-specific recommendations. In particular, the literature consistently highlighted blood supply chain resilience, network design, disruption management, and demand–supply coordination as critical determinants of crisis performance across diverse crisis contexts. As these aspects could not be sufficiently captured within the existing dimensions, Blood Supply Chain Design and Management was introduced as an additional standalone dimension. Furthermore, the original dimensions (Operations, Protocols, Resources, Communications, and Collaboration) were refined and expanded to reflect lessons learned across pandemics, natural disasters, armed conflicts, mass casualty incidents, and blood shortages. The resulting six-dimensional framework shifts the analytical perspective from crisis-specific recommendations toward a broader understanding of the organizational capabilities required to maintain preparedness, response capacity, operational continuity, and resilience. Although each dimension represents a distinct analytical perspective, multiple interdependencies exist between them. Together, the six dimensions provide a more comprehensive conceptual structure for understanding how blood donation services sustain operations under disruption and contribute to the three overarching operational practice themes identified in this review: coordinated governance and communication, operational continuity, and organizational resilience.
