# Supplementary material for: Lessons learned from crises in blood donation services: A systematic review of operational practices
Source: Vox Sang. 2026 Aug 20;121(10):1553–64. doi: 10.1111/vox.70337 (PMC13649367; doi:10.1111/vox.70337)
Supplement: Supplementary file 7 [file VOX-121-1553-s007.pdf]

Supplementary Table 8: Dimension 5 – Communication: Attributes and Practical Recommendations

	Attribute	Practical Recommendation	Attribute	Practical Recommendation
**Dimension 5: COMMUNICATION**	**Crisis Communication**	1. Establish dedicated crisis communication structures	**Digital Communication Strategies**	1. Leverage digital communication channels
		2. Implement clearly differentiated internal and external communication processes		2. Implement centralized digital communication and collaboration platforms
		3. Adapt communication strategies to the specific characteristics of each crisis		3. Utilize digital technologies for workforce training and competency development
	**Donor Communication**	1. Implement proactive and targeted donor communication strategies		
		2. Communicate clear, consistent, and transparent information on blood demand, donor safety, and infection prevention measures		
		3. Design communication campaigns that emphasize the societal value and lifesaving impact of blood donation		
