# Supplementary material for: Lessons learned from crises in blood donation services: A systematic review of operational practices
Source: Vox Sang. 2026 Aug 20;121(10):1553–64. doi: 10.1111/vox.70337 (PMC13649367; doi:10.1111/vox.70337)
Supplement: Supplementary file 8 [file VOX-121-1553-s008.pdf]

Supplementary Table 1: Crisis-Related and Blood Service-Related Keywords Used for Systematic Literature Search

Topic-Related Key Words	Sector-Related Key Words
disaster<br>crisis<br>resilien*<br>disrupt*<br>robust*	"Blood don*"<br>"Blood bank"<br>"Blood supply"<br>"Blood transfusion"
