# Supplementary material for: Lessons learned from crises in blood donation services: A systematic review of operational practices
Source: Vox Sang. 2026 Aug 20;121(10):1553–64. doi: 10.1111/vox.70337 (PMC13649367; doi:10.1111/vox.70337)
Supplement: Supplementary file 9 [file VOX-121-1553-s009.pdf]

Supplementary Table 5: Dimension 2 – Blood Supply Chain Design and Management: Attributes and Practical Recommendations

Dimension	Attribute	Practical Recommendation	Attribute	Practical Recommendation
**Dimension 2: BLOOD SUPPLY CHAIN DESIGN AND MANAGEMENT**	**Uncertainty and Disruption Management**	1. Strengthen supply chain visibility and forecasting capabilities	**Blood Collection and Distribution Management**	1. Develop decentralized and flexible collection and distribution networks
		2. Implement structured disruption management mechanisms		2. Integrate disruption risks, transportation capabilities, and demand–supply management into collection and distribution planning
		3. Leverage digital and technology-enabled solutions		3. Implement blood stewardship and donor flow management practices
	**Integrated and Adaptive Blood Supply Chains**	1. Adopt an integrated blood supply chain management approach	**Long-Term Planning and Supply Sustainability**	1. Implement long-term inventory management, forecasting, and demand-planning capabilities
		2. Strengthen adaptive planning and coordination among blood centres, healthcare providers, mobile collection facilities, and donor communities		2. Integrate the perishable nature of blood products into strategic planning processes
		3. Continuously monitor and manage interdependencies across the blood supply chain		3. Invest in advanced technologies, including artificial intelligence-based forecasting tools
