# Supplementary material for: Lessons learned from crises in blood donation services: A systematic review of operational practices
Source: Vox Sang. 2026 Aug 20;121(10):1553–64. doi: 10.1111/vox.70337 (PMC13649367; doi:10.1111/vox.70337)
Supplement: Supplementary file 10 [file VOX-121-1553-s010.pdf]

Supplementary Table 2: Overview of the inclusion and exclusion criteria

Inclusion Criteria	Exclusion Criteria
▪ Peer-reviewed journal articles<br>▪ Publications written in English<br>▪ Publications published within the last 15 years (2010-2025)<br>▪ Studies addressing blood donation services, blood banks, blood collection, blood supply, or transfusion service operations<br>▪ Studies examining crises, disasters, emergencies, disruptions, resilience, robustness<br>▪ Empirical, conceptual, review, modelling, or case-study publications reporting lessons learned, operational practices, experiences, recommendations, or management approaches.	▪ Non-peer-reviewed publications (e.g., editorials, commentaries, news articles, conference abstracts, dissertations)<br>▪ Publications not available in English.<br>▪ Publications outside the defined publication period<br>▪ Studies focusing exclusively on clinical, medical, immunological, or laboratory aspects without operational implications for blood donation services<br>▪ Studies unrelated to crises, disasters, disruptions, resilience, or emergency management<br>▪ Studies focusing exclusively on patient care, transfusion outcomes, or disease characteristics without implications for blood service operations<br>▪ Duplicate records.
